# Contamination by Aflatoxins B/G in Food and Commodities Imported in Southern Italy from 2017 to 2020: A Risk-Based Evaluation

**DOI:** 10.3390/toxins13060368

**Published:** 2021-05-22

**Authors:** Pasquale Gallo, Samantha Imbimbo, Silvana Alvino, Vincenzo Castellano, Olga Arace, Vittorio Soprano, Mauro Esposito, Francesco Paolo Serpe, Donato Sansone

**Affiliations:** Istituto Zooprofilattico Sperimentale del Mezzogiorno, 80055 Portici, Italy; samantha.imbimbo@izsmportici.it (S.I.); silvana.alvino@izsmportici.it (S.A.); vincenzo.castellano@cert.izsmportici.it (V.C.); olga.arace@cert.izsmportici.it (O.A.); vittorio.sopranow@gmail.com (V.S.); mauro.esposito@izsmportici.it (M.E.); francesco.serpe@izsmportici.it (F.P.S.); donato.sansone@izsmportici.it (D.S.)

**Keywords:** aflatoxins, food contamination, official control, risk evaluation

## Abstract

This study reports the results of aflatoxins B/G monitoring in food of vegetal origin, imported in Southern Italy from extra-European Union countries. From 2017 to 2020, we analyzed 1675 samples using an accredited HPLC method with fluorescence detection. We found out 295 samples (17.6%) were contaminated by aflatoxin B1, 204 by aflatoxins B/G (12.2%), while 75 (4.5%) resulted non-compliant to maximum limits set by the European Union law. Most of the batches tested were unprocessed food; the distribution of contamination levels, incidence of non-compliant samples, inference for different kinds of food are reported. The study focuses on the food more susceptible to contamination by aflatoxins; nuts are the food more controlled, showing the higher number of non-compliant samples. Our study confirms that pistachio nuts, hazelnuts and almonds are the major sources of exposure for consumers. Still, other products, such as chili pepper and Brazil nuts, need to get more information about their contamination levels. The study’s findings are discussed in the perspective of the last opinion by EFSA about chronic exposure to aflatoxins. A case study to evaluate not compliance of a composed food to the European Union law is reported.

## 1. Introduction

The contamination of food and feed by mycotoxins is a worldwide concern and a remarkable issue regarding food safety policies. Aflatoxins were discovered first in the 1960s, and their toxicity became increasingly evident in the 1970s and 1980s. Among mycotoxins, the aflatoxins (AFs) represent the most relevant risk to consumers because they are carcinogenic, genotoxic and teratogen compounds. They are the result of the secondary metabolism of toxigenic strains of the fungi *Aspergillus flavus* and *Aspergillus parasiticus* and can contaminate vegetation, cereals, nuts, as well as hay and feed. The origin of this contamination is natural, but it could also derive, or even be incremented, by improper storage of susceptible commodities (for example, chili peppers and other spices, nuts, rice, maize, wheat, figs, raisins, sesame seeds, sunflower seeds). This way, aflatoxins could be taken directly by consumers; moreover, contaminated feed can cause carryover in milk and derived products, such as cheeses. Both bioptic (climate changes, insect infestation) and unbioptic (hydro/thermal stress) factors affect aflatoxins’ presence in food. Anyway, for keeping mycotoxin levels as low as reasonably achievable and ensuring fair practices in the food trade, recommended good agricultural, storage and processing practices are necessary.

The International Agency for Research on Cancer (IARC) classified AFs as class 1 carcinogenic compounds to humans (IARC, 2002). Aflatoxin B1 (AFB1) is the most dangerous to both humans and animals;. However, AFB1 can be metabolized by the liver. Excessive exposure leads to acute liver necrosis and can cause cirrhosis or carcinoma (e.g., HCC hepatocellular carcinoma). The most widespread toxins are AFB1 and its metabolite aflatoxin B2 (AFB2), followed by aflatoxin G1 (AFG1) and its metabolite AFG2.

Only in 1995, the Codex Alimentarius Committee indicated for the first time that tolerable limits should be set for peanuts, almonds, shelled Brazil nuts, hazelnuts and pistachios intended for further processing [1], to protect consumers from the harmful effects of mycotoxins that may contaminate foodstuffs

In 2002, the European Commission (EC) laid down a Directive [2] to protect the consumers against exposure to contaminants, such as mycotoxins. In the same year, the European Commission started a community policy for food safety, the role of producers and the framework of the official controls of food and feed, which were completely defined and implemented within the Member States of the EU [3], including creating the European Food Safety Authority (EFSA). The EFSA must collect data about chemicals, contaminants and drugs in food and feed, evaluate risks for consumers and make proposals to the European Commission for improving food safety. Moreover, it was implemented the Rapid Alert System for Food and Feed (RASFF), a network covering the EU Member States to share real-time information, data and activities about food safety and related concerns, above all regarding foodstuffs and commodities from third countries. About aflatoxins in food, the European Commission issued in 2006 the requirements for sampling [4] and the maximum limits tolerable in food and commodities [5]. Both sampling procedures and maximum limits have been amended through time [6,7] based on the EFSA scientific opinions issued from 2007 [8] to 2020 [9]. For AFs in food, depending on the commodity and its destination of use, the EU set maximum tolerable limits for AFB1 (the most abundant and harmful) and for the sum of AFB1+AFB2+AFG1+AFG2 (total AF B/G). Italy is a remarkable importer of cereals, nuts, dried fruits from non-EU countries; our laboratory is involved in the official control of AFs in food and commodities entering four ports in the region Campania (Naples and Salerno) and the region Calabria (Gioia Tauro and Reggio Calabria), covering most of the imported commodities in Southern Italy. These official controls are carried out according to two criteria: the notifications/alerts from the RASFF and the monitoring control programs by the Italian Ministry of Health planned for controlling imported commodities on a statistical basis.

In 2017, the European Commission issued a new Regulation [10] regarding policies and activities for food safety, pointing out that official controls must be based on the risk analysis and risk assessment. To support the Italian public health authorities in planning controls, we studied the results of all the analyses performed between 2017 and 2020 in our laboratory regarding AFs B/G in food and commodities of vegetal origin. Herein we report this study to evaluate the percentage of contaminated samples and the levels of contamination, the inference for kind of product, the incidence of non-compliant food. A case study regarding applying EU regulations to evaluate a product’s compliance made of many ingredients is also described. This study gives an overview of the presence of aflatoxins in food largely consumed in Southern Italy. It allows focusing attention on some products that appear more susceptible to contamination, thus more harmful to consumers.

## 2. Results and Discussion

### 2.1. Sample Collection and Test Method

The samples tested were collected by the public authorities (USMAF) working in the ports of Naples and Salerno (region Campania), Gioia Tauro and Reggio Calabria (region Calabria). Sampling was performed according to the requirements of the EU law [4], as a follow-up of suspects and alerts from RASFF and random controls based on the amounts of imported commodities. Sampling was performed according to the guideline by the Italian Ministry of Health to apply the Regulation EC no. 401/2006 [4], issued to harmonize official controls. Accordingly, samples were collected depending on the amount of the batches, the kind of commodity, the packaging, using the dynamic or static procedure chosen by the competent authority in each case. All samples were placed in clean bags, closed and delivered to the laboratory; they were ground (dry or as a slurry, see Materials and Methods Section 4.4) and immediately analyzed. AFB1, AFB2, AFG1 and AFG2 were purified by immunoaffinity SPE chromatography, then separated and determined by reversed-phase HPLC with fluorescence detection (FLD) after photochemical derivatization by UVE. The test method was developed in-house and validated, then accredited according to the UNI EN ISO/IEC 17025 international standard (version 2018). About the test method, we measured the limit of quantification (LOQ) and calculated the limit of detection (LOD) for each aflatoxin (see Section 4.6). The HPLC-FLD method fits for the purposes of official control of food, according to the requirements of EU regulations for performance criteria. The purification by immunoaffinity, chromatographic separation and selective fluorescence detection, including post-column photo-derivatization, account for method specificity; the quality assurance controls run during each working session and using Shewhart control charts accounted for method trueness (mean recoveries) and within-laboratory reproducibility in terms of relative standard deviation (RSD).

### 2.2. Incidence and Levels of Contamination of AFB1 and the Sum AF B/G

**Figure 1:**
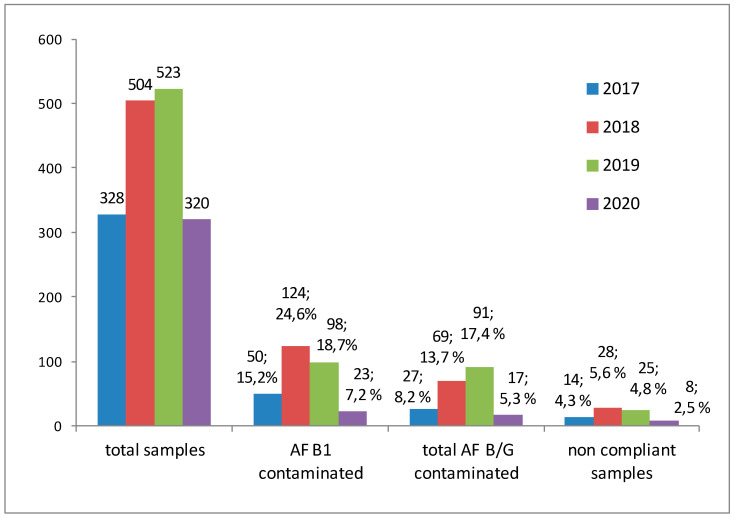
Samples analyzed each year, from 2017 to 2020, the number of those contaminated by both AFB1 and total AF B/G, the number of non-compliant commodities; for contaminated samples, the respective percentage are also reported.

The official control of aflatoxins in commodities from extra European Union countries entering the ports in Southern Italy increased from 2017 to 2019 (+59.4%) but diminished in 2020 because of the decrease in exportation due to the Sars-CoV2 pandemic (Figure 1). In this period, 1675 commodities were collected by local health authorities, then delivered to our laboratory and analyzed (Table 1). The nuts were largely the most controlled food (85.2% of total), followed by dried fruit (8.8%) and cereals and derivatives (3.1%). Almost in all kinds of food, we determined AFB1 and AF B/G in a significant percentage of samples. In the case of nuts, 17.8% and 12.5% of samples resulted contaminated by AFB1 and AF B/G, respectively; similar percentages have been observed in dried fruit, such as figs, dates, raisins (17.6% and 13.5%, respectively). In cereals (7.7% of samples) and hazelnut meals (22.2%), only AFB1 was determined.

Of course, increasing the control activities, we observed a major number of contaminated samples; addressing the controls makes them more effective and improves the protection of consumers. This is the role of the RASFF system, which makes it possible to share information in real time with the official control laboratories all over the European Union. The data show that AFB1 is the most abundant contaminant, present on 17.6% of all batches analyzed. Therefore, it needs particular attention for its harmfulness; the high rates of contaminated samples justify the focus of control activities on nuts and dried fruit. On the other hand, spices are quite susceptible to contamination by AFB1, and major attention is advisable, even in retail commodities; only 17 batches of chili pepper, pepper and paprika were delivered to the laboratory, but 23.5% of them was contaminated by AFB1. The highest content of AFB1 was determined at 27.4 µg/kg in the unique spice sample non-compliant, chili pepper, in 2017.

Bakery and pastry products included food destined for several uses as ingredients: 1 batch of seeds of sunflower from China for bakery, 4 kinds of wheat pasta, 1 cracker, 4 nut-based formulations for the ice cream industry. The 50.0% of samples resulted contaminated by AFB1 and AF B/G; they were all compliant to maximum limits. Thus these products do not seem particularly harmful to consumers, though pasta and crackers are very popular in Italy. The contamination levels in these products are relatively low; the highest concentrations were determined in a nut-based formulation to make ice cream, containing AFB1 at 3.1 µg/kg and the sum of total AF B/G at 5.3 µg/kg.

The single sample of vegetable extract collected was a hazelnut oil destined for pastry-making that was compliant with maximum limits.

**Figure 2:**
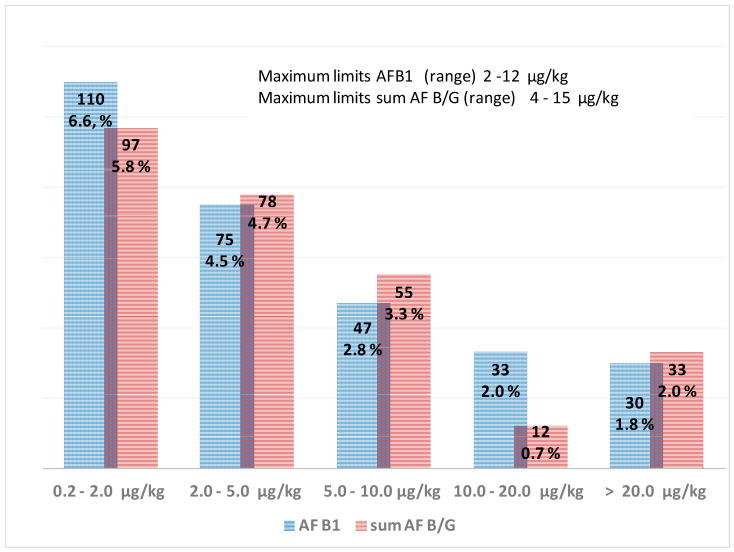
Distribution pattern for contamination levels of AFB1 and the sum AF B/G in the samples analyzed from 2017 to 2020; the relative percentages in respect to total sample number are also reported. The ranges for maximum limits for AFB1 and the sum AF B/G are shown.

The positive samples were grouped in concentration ranges to observe the contamination distribution pattern (Figure 2) compared to the maximum limits for AFB1 and the sum AF B/G in different commodities (Table 2). The maximum limits span from 2.0 to 12.0 µg/kg for AFB1 and from 4.0 to 15.0 µg/kg for the sum of total AFB/G [5,6]. The Regulation EC no. 1881/2006 stated these maximum limits apply to the food as described in Table 2; for commodities that were processed (roasting, dilution, salting, desiccation) and compound foodstuffs, the specific dilution or concentration factors must be evaluated to assess sample compliance. This makes it more difficult to control retail commodities; most of the food and commodities considered for this study were not processed. In many cases, we had to apply dilution or concentration factors to assess compliance. In Figure 2, the distribution of contamination levels due to AFB1 shows a polynomial (order 2) trend (R^2^ = 0.9993). Similar behavior can be observed for contamination by the sum AF B/G (R^2^ = 0.8692). For concentrations above 20.0 µg/kg, we did not calculate ranges for contamination distribution because they were quite higher than the maximum upper limit at 15 µg/kg; this way, we would give an immediate view of the trend in samples exceeding the maximum limits. Regarding AFB1, in some cases, the concentrations determined were very high; particularly, we found out 8 batches of pistachio nuts containing AFB1 between 58 and 135 µg/kg (from about 5 to 11-fold the maximum limits), 1 peanut sample for direct human consumption contaminated at 59 µg/kg (about 30-fold the maximum limit). Still, the highest content was determined in dried figs at 255 µg/kg (fifty-one times the maximum limit).

### 2.3. Non-Compliant Samples

The EU law requires that the compliance of food and commodities must be decided concerning the maximum limits for AFB1 and/or the sum of AFB1+AFB2+AFG1+AFG2 (Table 2), considering the measurement uncertainty. Over the whole period studied, 75 samples out of 1675 were non-compliant (4.5%), that is, the concentration of AFB1 or the sum AF B/G, or both were above their respective maximum limits, even taking into account the measurement uncertainty (Table 3). All non-compliant samples exceed the maximum limit for AF B1. In some cases, also for the sum of total AF B/G; as a matter of fact, AFB1 is the major contaminant and the main risk for consumers. Apart from 2020, when fewer samples were analyzed, the rate of non-compliant samples was higher than 4% every year. In Table 3, the non-compliant samples per year and per different kinds of food/commodities are reported. Pistachio nuts (52.0% of total non-compliant), hazelnuts (13.3%) and almonds (12.0%) are the food that in more cases were rejected for not compliance to maximum limits; the results confirm that in Southern Italy, imported nuts represent the major risk for consumers referring to the contamination by aflatoxins B/G. The samples of nuts delivered to the laboratory were both shelled and unshelled, never a mixture; regardless, the EU laws were applied for sampling, grinding and calculation of results. The percentage of non-compliant for different food and commodities over 2017–2020 was also calculated (Table 4). Several batches of pistachio nuts were controlled because of several RASFF alerts, showing a 10.6% non-compliant rate; on the contrary, peanuts showed a lower rate of non-compliant batches (1.4%), despite 484 controls were carried out. Instead, relatively high rates of non-compliant were found out regarding Brazil nuts (23.1%) and chili peppers (12.5%), although only a few batches were tested in these years (13 and 8, respectively). Furthermore, for almonds, a significant rate of non-compliant samples was observed (4.0%), controlling 225 batches. Regarding cereals, a few controls were carried out (15), depending on the low amounts of commodities entering the ports considered for this study; only 1 batch of rice was non-compliant.

The annual reports from the RASFF [11,12,13] show that these findings are coherent with the notifications and alerts from the network system in the last years; aflatoxins in nuts, nut products and seeds are always at the top of the number of notifications/alerts, from most EU Member States. Indeed, every year the highest number of rejections of commodities at the borders of the EU Member States is due to not compliance with aflatoxins. Non-compliant products object of notifications/alerts from the RASFF were above all from Turkey, Northern and Southern America, China, Egypt, Iran. Among the Member States, Italy is one of the most active in the detection of non-compliant food/commodities, and the monitoring by official control laboratories is effective to prevent contaminated products enter the production processes

### 2.4. Comparison of Contamination Levels and Maximum Limits for Risk Evaluation

**Figure 3:**
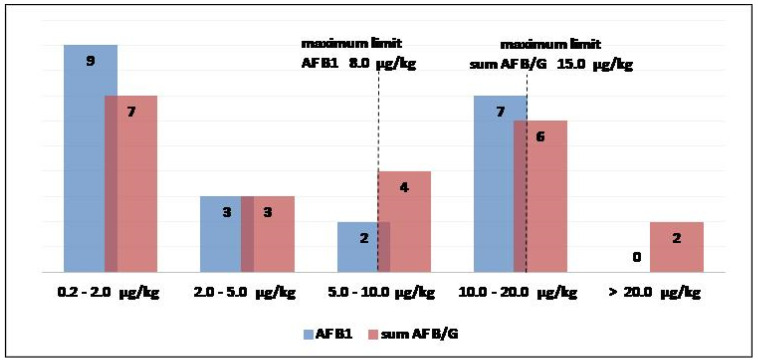
Distribution patterns for contamination levels of AFB1 and the sum AF B/G in the 180 batches of hazelnut and hazelnut meal analyzed from 2017 to 2020. The dashed lines represent the maximum limits for AFB1 and the sum AF B/G in products not intended for direct human consumption.

The data can also be evaluated for single food/commodity. We report the case of hazelnut and hazelnut meal because these products are often consumed directly (raw, peeled, roasted), above all in winter, but are also largely used to prepare chocolate creams and ice creams for important Italian brands, as well as in the pastry-making industry, which is considerable for consumers in Southern Italy. From 2017 to 2020, the health authorities collected 180 batches, above all for monitoring programs; as a result, 21 batches were contaminated by AFB1, and 22 by the AF B/G (Figure 3). The maximum limits (at 8.0 and 15.0 µg/kg for AF B1 and the sum AF B/G, respectively) for hazelnut not intended for direct human consumption are reported in Figure 3 to show immediately non-compliant in respect to the contaminated samples. Aflatoxins, like other mycotoxins, cause heterogeneous contamination in food; for the hazelnut we analyzed, the patterns of AFB1 and sum of AFB/G contamination distributions show the maximum limits can be exceeded in several cases, with no apparent trend. The controls of commodities from non-EU countries aim to prevent using non-compliant products, but the tolerable contamination levels and the large consumption of this food require an evaluation of risks to consumers due to the exposure to AFB/G. The last Scientific Opinion from the EFSA [9] confirmed that liver carcinogenicity of aflatoxins remains the pivotal effect for the risk assessment, based on an estimation made by the Joint FAO/WHO Expert Committee on Food Additives (JECFA) in 2016. Moreover, the Scientific Opinion stated that risk assessments will consider AFB1, AFB2, AFG1 and AFG2, with particular attention to the chronic dietary exposure. The monitoring activity for an estimation, using a data set comprising 209.802 analytical results from 69.166 samples, pointed out the highest AFB1 and total AFB/G mean concentrations were obtained for the food category “legumes, nuts and oilseeds” (in particular for pistachios, peanuts and other seeds). The exposure of consumers should be evaluated based on the relationship between biomarker levels and dietary intake at the individual level; to this aim, data about indigenous dietary exposure to aflatoxins for groups of populations are necessary.

A few surveys have been published in the last years about food contaminated by aflatoxins. Regarding Italy, Imperato et al. [14] described in 2011 the results of 345 samples of several foods imported in Italy; they found out 7% of samples were contaminated by total AFs, above all hazelnut paste, but only 1.2% of batches were non-compliant. The highest concentration at 70.69 μg/kg was determined in apricot kernels. In 2012 a survey reported the levels of contamination by AFs in dried chestnuts (37) and chestnut flour samples (14) produced in Italy [15]; the authors found out these food are largely contaminated by AFB1 (62.2 and 21.4% in chestnut flour and dried chestnuts, respectively). Many samples were non-compliant (24.3% and 7.1%, respectively). A high incidence of AFG1 was also observed (40.5%). The highest concentrations forAFB1 and the sum of total AF B/G were 67.88 and 188.78 μg/kg, respectively, in the chestnut flour sample. In the same year, the occurrence of AFB1 in conventional and organic flour from the retail market in Italy was described [16]. Several samples were tested (20 conventional wheat flour, 20 organic wheat flour, 42 conventional corn flour, and 8 organic corn flour). AFB1 was detected in 13 samples of corn flour (4 organics and 9 conventional), confirming a higher incidence of contamination in corn than wheat. The levels of contamination ranged between 0.17 and 3.75 μg/kg. Just one sample was non-compliant.

In the other EU Member States, a few surveys are available. In 2008 O’Riordan et al. [17] reported a survey about AFs contents of 130 commercial spice preparations, including pepper, chili, curry powder, cayenne, paprika, cinnamon, coriander, turmeric and cumin, from retail markets in Ireland. They observed AFB1 highest incidence in spice preparations (20 out of 130 samples were contaminated), up to 27.5 μg/kg in a sample of chili powder; five samples (3.8%), consisting of chili, cayenne pepper and turmeric pepper, were non-compliant. In 2010 Reiter et al. [18] reported the results about AFs contents in 81 rice samples from different markets in Vienna. They determined AFB1 in 15 out of 81 samples, in the range 0.45–9.86 μg/kg; just 3 samples were non-compliant.

### 2.5. A Case Study for Compliance Assessment: A Mix of Dried Nuts

In 2019, the health authority USMAF sampled in the port of Gioia Tauro (Calabria) a batch of a product for direct human consumption from Syria. The product was a mix of nuts (roasted pistachio nuts, roasted peanuts, roasted almonds, cashew nuts), chickpea and pumpkin seeds; the product was imported pre-dosed in large packages. For this product, no maximum limit has been set by the European Union legislation. Referring to the art. 3 of the Regulation EC no. 1881/2006 [5], to perform the analysis, we physically separated all the ingredients of the mix; chickpeas were not tested because no legal limit is available, while all the other ingredients were analyzed separately. The single results compared to the respective maximum limits for commodities intended for direct human consumption (Table 5).

We found out the ingredients tested were not contaminated, apart from the roasted peanuts, at concentrations exceeding the maximum limits for both AFB1 and the sum of total AF B/G. The product was non-compliant, thus was rejected. In this case, the direct analysis of the mix as a whole product is not possible because dilution or concentration factors cannot be applied to this composed product. This is an emblematic case because the solution was in the requirement that non-compliant contaminated ingredients cannot be used to produce food and commodities and must be avoided before the manufacturing process; such a problem could be observed when controlling retail commodities.

## 3. Conclusions

A risk-based analysis is mandatory in the EU States to address effective public controls and guarantee food safety; a milestone of the EU policy for food safety is to reduce exposure of consumers to contaminants, such as aflatoxins, considered among the most harmful. The monitoring of AF B/G in food and commodities imported in Southern Italy from the extra EU States between 2017 and 2020 confirmed that nuts are the most relevant source of exposure for consumers to aflatoxins. In particular, although only a few samples showed very high concentrations of AFB1 and total AFB/G, for pistachio nuts and hazelnuts, the monitoring of contamination levels appears necessary; the data should be used to evaluate chronic exposure of a group of consumers, depending on consumption habits. The coherence of our data with the overall results in the RASFF reports about the last three years indicates the network system for the rapid alert is highly effective in preventing the risks from contamination due to aflatoxins. This study highlights the need to control raw materials to prevent contamination of food. Anyway, the results suggest also that other food, like chili pepper and Brazil nuts, should be controlled to get more insights into contamination levels. The case of a mix of dried nuts we described points out the problems in controlling retail compound products and assessing compliance. Finally, bakery and pastry products were poorly controlled. Still, the rate of non-compliant samples (22.2%) suggests more information about AFB/G contamination is necessary.

## 4. Materials and Methods

### 4.1. Chemicals and Reagents

HPLC grade methanol and acetonitrile were purchased from Sigma-Aldrich (Milan, Italy); HPLC grade water was in-house produced by a Milli-Q system (Sartorius, Goettingen, Germany).

The reference standard materials AFB1, AFB2, AFG1 and AFG2 in acetonitrile solutions, at 100 µg/mL, were supplied by Sigma-Aldrich (Milan, Italy).

AflaTest™ WB immunoaffinity fast flow disposable columns were used to purify AFs from food (VICAM, Boston, MA, USA).

### 4.2. Working Standard Solutions

A standard mix solution at 1.0 µg/mL of each aflatoxin was prepared from reference standard materials at 100 µg/mL by dilution in acetonitrile. A working standard mix solution at 20.0 ng/mL was daily prepared by diluting the standard mix solution at 1.0 µg/mL with methanol. Calibration standard mix solutions at 0.31, 0.62, 1.25, 2.5, 5.0 and 10.0 ng/mL of each aflatoxin were prepared by diluting appropriately in methanol the 20.0 ng/mL standard mix solution. For quantitative analysis, external standard linear regression calibration curves were calculated during each working session, injecting the mix of AF B/G standards twice.

### 4.3. Quality Assurance Controls of the Analytical Processes

During each working session, blank reagents and one blank process were run. Moreover, AF recoveries were verified using black samples spiked with AFB1, AFB2, AFG1 and AFG2 at 10.0 µg/kg (for cereals, nuts, dried fruit, oilseeds, bakery and pastry products, vegetable extracts) and 5.0 µg/kg (for spices and herbs, hazelnut meals), to evaluate trueness (mean recoveries) and within-laboratory reproducibility (in terms of standard deviation, SD_R_). To monitor the analytical performance through analyses, we record the recoveries of both AFB1 and of the sum total AF B/G by Shewhart control charts reporting the mean recoveries calculated during the first validation studies and the correlated within-laboratory reproducibility (SD_R_), in the ranges ± SD_R_, ± 2 SD_R_, ± 3 SD_R_.

For samples spiked at 10.0 µg/kg, the mean value and SD_R_ is (8.81 ± 0.83) µg/kg for AF B1, and (8.45 ± 0.84) µg/kg for the sum total AF B/G.

In the case of samples spiked at 5.0 µg/kg, the mean value and SD_R_ is (4.95 ± 0.71) µg/kg for AF B1, and (3.51 ± 0.22) µg/kg for the sum total AF B/G.

### 4.4. Sample Clean Up

According to Regulation EC no. 401/2006 and the guideline from the Italian Ministry of Health, the samples collected can be homogenized dry and finely, or can be mixed to water in a 1:1 ratio, then ground to form a slurry. We prepared slurry for all kinds of food/commodities, apart from figs, pepper and chili pepper. The portion for the analysis was taken immediately, and the remaining part was stored at −18 °C. For dry samples, 25.00 ± 0.01 g were weighed in a 250 mL bottle, added whit 5.0 g of NaCl and 125 mL of the extraction solution methanol/H_2_O 60/40 (*v*/*v*) (dried fruit) or 125 mL of methanol (spices and potherbs).

For samples homogenized as slurry, 50.00 ± 0.01 g were weighed in a 250 mL bottle, added with 5.0 g of NaCl and 100 mL of the methanol/H_2_O 75/25 (*v*/*v*) solution.

The sample was mixed on a horizontal shaker for 3 min, centrifuged at 2500 *g* for 5 min at 4 °C; the supernatant was filtered through Whatman n. 41 paper filter and collected in a 250 mL glass bottle. The sample was extracted again using 125 mL of HPLC grade water; then was mixed, centrifuged, and the supernatant filtered and combined with the previous extract. Then, 10.0 mL of extract was loaded by gravity onto the immunoaffinity column (IAC), which was washed with 10 mL HPLC grade water; finally, the AFs were eluted from IAC by 2.0 mL methanol at a flow rate of approximately 2–3 drops/second. The eluate sample collected in a test tube was ready for HPLC-FLD analysis.

### 4.5. HPLC-FLD Determination after Photochemical Derivatization

AFs were determined by an HPLC system Agilent 1200 Series, equipped with a fluorescence detector (FLD) Agilent 1100 Series, an autosampler and variable loop automatic injector. Chromatographic separation was performed on a 250 × 4.6 mm Synergi Polar-RP80 reversed-phase stainless steel column, particle 4 µm (Phenomenex, Italy). An online photochemical reactor (UVE, LC Thenen, Obertaufkirchen, Germany) was used for the post-column derivatization of AFB1 and AFG1, which do not fluoresce under standard reversed-phase HPLC conditions.

The mobile phase was water/acetonitrile/methanol (53:23:24, *v*/*v*/*v*) in isocratic mode at a flow rate of 1.0 mL/min. The column oven set at 25 °C; the injection volume was 100 µL.

The total runtime of each analysis was 32 min, and the retention times of the AFs were approximately 15.0 min for AFG2, 18.5 min for AFG1, 20.0 min for AFB2 and 25.0 min for AFB1.

For the detection of AFs, excitation and emission wavelengths were set at 365 nm and 435 nm, respectively.

For quantitative determination, seven-point standard calibration curves were calculated by linear regression for each aflatoxin by plotting the chromatographic peak area of the calibration standard versus the concentration. The presence of aflatoxins in the samples was confirmed by comparing the retention times of peaks with those of the calibration standards. The concentrations of AFs were reported in µg/kg of sample. The method was in-house validated for all the kinds of food/commodity tested, according to the performance criteria of the EU regulations, and is accredited according to the UNI EN ISO/IEC 17025:2018 international standard.

### 4.6. Method Validation and Performance

The HPLC-FLD method was validated according to the procedure of our quality management system to comply with the performance criteria required by Regulation EC no. 401/2006 [4] and Regulation EC no. 882/2004 [19]. We assessed the specificity for all the matrices tested, verifying there was no interference in the HPLC-FLD chromatograms. The linearity of the detector response expressed as correlation value R^2^ was measured by external standard linear regression calibration curves, calculated during each working session. Method trueness was evaluated as mean recoveries, analyzing different samples spiked at two or three levels of contamination of AFB1, AFB2, AFG1, AFG2. The same samples were used to calculate method precision in terms of repeatability (as relative standard deviation, RSD_r_, at each level, in the same day) and within-laboratory reproducibility (as relative standard deviation, RSD_R_, at each level, in different days, different analysts). The LOQs for each AF were calculated according to the procedures of our quality management system, as the lowest concentrations that were measured analyzing real uncontaminated samples (different nuts, cereals, spices, figs) spiked at decreasing amounts, showing a signal-to-noise/ratio of 10:1. The corresponding LODs were calculated from the lowest standard of the calibration curves. The data are summarized in Table S1 as supporting info.

## Figures and Tables

**Table 1:** Food/commodities from extra-European Union countries analyzed in 2017–2020; the number of samples contaminated by both AFB1 and total AF B/G, and the respective percentages, are reported.

	Contaminated Samples				
Food/Commodity	Total Sample Number	AF B1	Total AF B/G	% AF B1	% Total AF B/G
Nuts (hazelnuts, groundnuts, almonds, walnuts, pistachio nuts, peanuts, pecans, cashews)	1427	254	178	17.8	12.5
Dried fruit (figs, dates, raisins)	148	26	20	17.6	13.5
Cereals and derivatives (rice, wheat, maize)	52	4	0	7. 7	0.0
Spices (chili peppers, pepper, paprika)	17	4	1	23.5	5.9
Oilseeds and derivatives	11	0	0	0.0	0.0
Bakery and pastry products (sunflower seeds, pasta, crackers, nut-based formulations)	10	5	5	50.0	50.0
Hazelnut meal	9	2	0	22.2	0.0
Vegetable extracts (hazelnut oil)	1	0	0	0.0	0.0
Total samples	1675	295	204	17.6	12.2

**Table 2:** Maximum tolerable limits for AF B1 and the sum total AF B/G in different foods/commodities.

	Maximum Limits (µg/kg)	
Food/Commodity	AFB1	Sum of AFB1, AFB2, AFG1, AFG2
Groundnuts (peanuts) and other oilseeds to be subjected to sorting, or other physical treatment, before human consumption or use as an ingredient in foodstuffs with the exception of Groundnuts (peanuts) and other oilseeds for crushing for refined vegetable oil production	8.0	15.0
Almonds, pistachios and apricot kernels to be subjected to sorting, or other physical treatment, before human consumption or use as an ingredient in foodstuffs	12.0	15.0
Hazelnuts and Brazil nuts, to be subjected to sorting, or other physical treatment before human consumption or use as an ingredient in foodstuffs	8.0	15.0
Tree nuts, other than the tree nuts listed in 2.1.2 and 2.1.3, to be subjected to sorting, or other physical treatment, before human consumption or use as an ingredient in foodstuffs	5.0	10.0
Groundnuts (peanuts) and other oilseeds and processed products thereof, intended for direct human consumption or use as an ingredient in foodstuffs, with the exception of Crude vegetable oils destined for refining; Refined vegetable oil.	2.0	4.0
Almonds, pistachios and apricot kernels, intended for direct human consumption or use as an ingredient in foodstuffs	8.0	10.0
Hazelnuts and Brazil nuts, intended for direct human consumption or use as an ingredient in foodstuffs	5.0	10.0
Tree nuts, other than the tree nuts listed in 2.1.6 and 2.1.7, and processed products thereof, intended for direct human consumption or use as an ingredient in foodstuffs	2.0	4.0
Dried fruit to be subjected to sorting, or other physical treatment, before human consumption or use as an ingredient in foodstuffs	5.0	10.0
Dried fruit and processed products thereof, intended for direct human consumption or use as an ingredient in foodstuffs	2.0	4.0
All cereals and all products derived from cereals, including processed cereal products, with the exception of maize and foodstuffs listed in 2.1.12 and 2.1.17	2.0	4.0
Maize and rice to be subjected to sorting or other physical treatment before human consumption or use as an ingredient in foodstuffs	5.0	10.0
Following species of spices: *Capsicum* spp. (dried fruits thereof, whole or ground, including chilies, chili powder, cayenne and paprika); *Piper* spp. (fruits thereof, including white and black pepper); *Myristica fragrans* (nutmeg); *Zingiber officinale* (ginger); *Curcuma longa* (turmeric); Mix of spices containing one or more of the previous spices.	5.0	10.0

**Table 3:** The non-compliant samples between 2017 and 2020, per year and kind of food/commodity; the respective incidences versus the total number of non-compliant samples are reported.

Food/Commodity	2017	2018	2019	2020	Non-Compliant Samples
Pistachio nuts	5	15	13	6	39 (52.0%)
Hazelnuts	4	3	2	1	10 (13.3%)
Almonds	2	5	2	0	9 (12.0%)
Peanuts	0	2	4	1	7 (9.3%)
Dried figs	2	0	2	0	4 (5.3%)
Brazil nuts	0	2	1	0	3 (4.0%)
Chili peppers	1	0	0	0	1 (1.3%)
Mix of dried nuts	0	0	1	0	1 (1.3%)
Rice	0	1	0	0	1 (1.3%)
TOTAL	*14*	*28*	*25*	*8*	*75*
% Non-compliant samples out of total samples	*4.3%*	*5.6%*	*4.8%*	*2.5%*	*4.5%*

**Table 4:** Non-compliant samples in 2017–2020 per kind of food/commodity and the respective incidences.

Food/Commodity	Total Non-Compliant Samples per Food/Commodity	Samples 2017–2020	% Non-Compliant Samples per Food/Commodity
Pistachio nuts	39	369	10.6%
Hazelnuts	10	180	5.6%
Almonds	9	225	4.0%
Peanuts	7	484	1.4%
Dried figs	4	147	2.7%
Brazil nuts	3	13	23.1%
Chili peppers	1	8	12.5%
Mix of dried nuts	1	1	100.0%
Rice	1	15	6.7%

**Table 5:** Mix of dried fruit products for direct human consumption analyzed after physical separation of all the ingredients to assess not compliance referring to respective maximum limits.

Mix Ingredients	Ingredient	Results		Maximum Limits (ML). µg/kg	
	% in Weight	µg/kg		AF B1	Sum of AF B/G
Roasted pistachio nut	9.8%	<LOQ	<LOQ	8.0	10.0
Roasted peanuts	51.8%	48.2 ± 4.9	80.4 ± 5.9	2.0	4.0
Roasted almonds	9.5%	<LOQ	<LOQ	8.0	10.0
Cashew nuts	10.4%	<LOQ	<LOQ	5.0	10.0
Pumpkin seeds	9.1%	<LOQ	<LOQ	2.0	4.0
Chickpeas	9.4%	Not analyzed	Not analyzed	No ML	No ML

## Data Availability

All data supporting this study are preserved at the Istituto Zooprofilattico Sperimentale del Mezzogiorno, in Portici (NA), Italy.

## Supplementary Materials

The following are available online at https://www.mdpi.com/article/10.3390/toxins13060368/s1, Table S1: The results of validation studies for determination of AF B/G in several food.
