# Supplementary material for: Contamination by Aflatoxins B/G in Food and Commodities Imported in Southern Italy from 2017 to 2020: A Risk-Based Evaluation
Source: Toxins (Basel). 2021 May 22;13(6):368. doi: 10.3390/toxins13060368 (PMC8224785; doi:10.3390/toxins13060368)
Supplement: Supplementary file 1 [file toxins-13-00368-s001.zip › toxins-1225672-supplementry.pdf]

# Supplementary Materials: Contamination by Aflatoxins B/G in Food and Commodities Imported in Southern Italy from 2017 to 2020: A Risk-Based Evaluation

**Pasquale Gallo, Samantha Imbimbo, Silvana Alvino, Vincenzo Castellano, Olga Arace, Vittorio Soprano, Mauro Esposito, Francesco Paolo Serpe and Donato Sansone**

**Table S1.** The results of validation studies for determination of AF B/G in several food.

| Food | Validation Parameter | AF B1 | AF B2 | AF G1 | AF G2 |
|---|---|---|---|---|---|
| nuts, cereals, dried fruit oilseeds | trueness, mean recoveries at 1,0 µg/kg | 101,70% | 102,80% | 100,30% | 98,30% |
| | trueness, mean recoveries at 5,0 µg/kg | 87,70% | 97,70% | 93,50% | 88,90% |
| | trueness, mean recoveries at 15,0 µg/kg | 90,20% | 96,00% | 92,70% | 91,80% |
| | repeatability at 1,0 µg/kg , $RSD_r$, n = 6 | 3,50% | 5,70% | 6,90% | 5,40% |
| | repeatability at 5,0 µg/kg , RSDr, n = 6 | 3,80% | 1,40% | 1,80% | 6,10% |
| | repeatability at 15,0 µg/kg ,RSDr, n = 6 | 4,90% | 1,10% | 1,70% | 3,30% |
| | within-laboratory reproducibility at 1,0 µg/kg , $RSD_R$, n = 16 | 6,80% | 5,70% | 6,00% | 10,80% |
| | within-laboratory reproducibility at 5,0 µg/kg , $RSD_R$, n = 16 | 8,00% | 11,60% | 6,60% | 14,70% |
| | within-laboratory reproducibility at 10,0 µg/kg , $RSD_R$, n = 16 | 6,90% | 11,20% | 5,80% | 10,30% |
| spices, potherb | trueness, mean recoveries at 2,0 µg/kg | 93,50% | 87,40% | 84,40% | 67,90% |
| | trueness, mean recoveries at 5,0 µg/kg | 99,00% | 91,80% | 90,50% | 70,20% |
| | trueness, mean recoveries at 15,0 µg/kg | 98,10% | 93,60% | 98% | 78,30% |
| | repeatability at 2,0 µg/kg , $RSD_r$, n = 6 | 16,00% | 13,70% | 8,60% | 13,30% |
| | repeatability at 5,0 µg/kg , RSDr, n = 6 | 16,10% | 15,30% | 12,00% | 14,00% |
| | repeatability at 15,0 µg/kg ,RSDr, n = 6 | 12,40% | 17,80% | 9,80% | 10,50% |
| | within-laboratory reproducibility at 2,0 µg/kg , $RSD_R$, n = 12 | 12,80% | 11,50% | 8,20% | 10,90% |
| | within-laboratory reproducibility at 5,0 µg/kg , $RSD_R$, n = 12 | 14,30% | 14,60% | 11,00% | 12,50% |
| | within-laboratory reproducibility at 15,0 µg/kg , $RSD_R$, n = 12 | 10,80% | 15,10% | 8,20% | 7,60% |
| bakery products, vegetable extracts | trueness, mean recoveries at 0,25 µg/kg | 113,30% | 100,00% | 106,70% | 67,30% |
| | trueness, mean recoveries at 2,0 µg/kg | 95,80% | 90,70% | 93,80% | 95,00% |
| | trueness, mean recoveries at 4,0 µg/kg | 98,90% | 99,10% | 96,10% | 90,20% |
| | repeatability at 0,25 µg/kg , $RSD_r$, n = 6 | 5,30% | 9,10% | 5,10% | 8,70% |
| | repeatability at 2,0 µg/kg , RSDr, n = 6 | 5,90% | 4,00% | 9,00% | 7,30% |
| | repeatability at 4,0 µg/kg ,RSDr, n = 6 | 6,10% | 9,80% | 4,70% | 7,80% |
| hazelnut meals | trueness, mean recoveries at 1,0 µg/kg | 90,30% | 84,80% | 97,30% | 75,70% |
| | trueness, mean recoveries at 15,0 µg/kg | 91,00% | 91,40% | 96,90% | 83,70% |
| | repeatability at 1,0 µg/kg , $RSD_r$, n = 6 | 3,00% | 5,80% | 7,70% | 6,40% |
| | repeatability at 15,0 µg/kg ,RSDr, n = 6 | 3,70% | 2,30% | 2,80% | 8,20% |

| | | | | | |
|---|---|---|---|---|---|
| | within-laboratory reproducibility at 1,0 μg/kg , $RSD_R$, n = 8 | 2,90% | 7,90% | 10,10% | 12,60% |
| | within-laboratory reproducibility at 15,0 μg/kg , $RSD_R$, n = 8 | 3,30% | 5,50% | 4,30% | 20,60% |
| cereals, nuts, dried fruit, oilseeds | LOQ, μg/kg | 0,25 | 0,25 | 0,25 | 0,25 |
| bakery products, vegetable extracts | LOD, μg/kg | 0,25 | 0,25 | 0,25 | 0,25 |
| spices, potherb | LOQ, μg/kg | 2,0 | 2,0 | 2,0 | 2,0 |
| | LOD, μg/kg | 0,42 | 0,42 | 0,42 | 0,42 |
| hazelnut meals | LOQ, μg/kg | 1,0 | 1,0 | 1,0 | 1,0 |
| | LOD, μg/kg | 0,25 | 0,25 | 0,25 | 0,25 |
| all food | linearity of detector signal, standard calibration curve $R^2$ | ≥ 0,99 | ≥ 0,99 | ≥ 0,99 | ≥ 0,99 |
